# Involvement of Oxidative Stress and the Epidermal Growth Factor Receptor in Diesel Exhaust Particle-Induced Expression of Inflammatory Mediators in Human Mononuclear Cells

**DOI:** 10.1155/2019/3437104

**Published:** 2019-11-15

**Authors:** Fangfang Li, Zhen An, Haibin Li, Xia Gao, Gui Wang, Weidong Wu

**Affiliations:** ^1^Department of Industrial and Environmental Health, Xinxiang Medical University, Xinxiang, Henan Province 453003, China; ^2^Department of Health Inspection and Quarantine, School of Public Health, Xinxiang Medical University, Xinxiang, Henan Province 453003, China

## Abstract

Exposure to diesel exhaust particles (DEPs) has been associated with increased incidence of cardiopulmonary diseases. This study is aimed at examining the proinflammatory effects of DEP on primary human peripheral blood mononuclear cells (PBMC) and the underlying mechanisms using a human mononuclear cell line, THP-1. DEPs were incubated with the PBMC and THP-1 cells for 24 h, respectively. The supernatants were collected and subjected to measurement of proinflammatory mediators including interleukin 8 (IL-8) or tumor necrosis factor *α* (TNF*α*) by ELISA. Levels of reactive oxygen species (ROS) were determined using flow cytometry. Phosphorylation of the epidermal growth factor receptor (EGFR) was examined with immunoblotting. Exposure to DEP induced a concentration-dependent increase in the expression of IL-8 and TNF*α* in the PBMC and THP-1 cells. Further mechanistic studies with THP-1 cells indicated that DEP stimulation increased intracellular levels of ROS, an indicator of oxidative stress, and phosphorylation of the EGFR, indicative of EGFR activation. Pretreatment of THP-1 cells with the antioxidant N-acetyl-L-cysteine (NAC) markedly blunted DEP-induced EGFR phosphorylation, indicating that oxidative stress was involved in DEP-induced EGFR activation. Furthermore, the pretreatment of THP-1 cells with either NAC or a selective EGFR inhibitor significantly blocked DEP-induced IL-8 expression, implying that oxidative stress and subsequent EGFR activation mediated DEP-induced inflammatory response. In summary, DEP stimulation increases the expression of proinflammatory mediators in human mononuclear cells, which is regulated by oxidative stress-EGFR signaling pathway.

## 1. Introduction

Exposure to air pollution, especially airborne particulate matter (PM), has been associated with increased morbidity and mortality for cardiopulmonary diseases [1–6]. Based on aerodynamic diameter, PM can be classified into coarse (2.5-10 *μ*m, PM_2.5-10_), fine (<2.5 *μ*m, PM_2.5_), and ultrafine (<0.1 *μ*m, PM_0.1_) particles. Of them, PM_2.5_ and PM_0.1_ can be inhaled more deeply into the lungs with a portion depositing in the alveoli and entering the pulmonary circulation and likely the systemic circulation. Source apportionment study on the main categories of PM has indicated that, on average, traffic is the biggest source of air pollution in urban air around the world, responsible for one quarter of PM in the air [7]. The associations of air pollution with cardiopulmonary diseases are the strongest for PM_2.5_, of which the combustion-derived ultrafine particles from diesel exhaust are an important component [2].

Diesel engines are cheaper to run than gasoline engines or other sources of power. However, diesel combustion can emit a complex mixture of air pollutants, including both gaseous and solid material. The solid material mainly refers to diesel exhaust particles (DEPs), which account for a highly significant percentage of the smallest sizes of PM emitted in urban areas. For example, a study with electron microscopy showed that over 80% of DEP had a size ≤ 0.1 *μ*m [8]. Given the significant contribution of PM emissions from diesel vehicles to the total concentration of PM in ambient air, much research has been carried out on effects of DEP that have been used as the surrogate in exploring the mechanisms by which airborne fine or ultrafine particles promote cardiopulmonary diseases [9]. Thus far, the exact causal connection between DEP and adverse health effects is still not fully understood, but certain molecular and cellular mechanisms are generally assumed to play a key role. The most well-examined cellular responses upon interaction with DEP are the induction of oxidative stress and inflammation, both of which play a key role in the onset or exacerbation of respiratory diseases and the development of cardiovascular diseases [10]. Acute coronary syndrome (ACS) is a syndrome due to decreased blood flow in the coronary arteries such that part of the heart muscle is unable to function properly or dies [11]. Previous study has shown that mortality from all causes was higher among individuals with greater long-term exposure to PM_2.5_ in survivors of hospital admission for ACS [12]. Elevated PM_2.5_ exposure contributes to triggering acute coronary events, especially ST-segment elevation myocardial infarction, in those with existing seriously diseased coronary arteries but not in those with nondiseased coronary arteries [13]. These results suggest that ACS patients are susceptible to PM_2.5_ effects. There is extensive evidence to support a pathogenic role for both local and systemic inflammation in ACS, and the intensity of the inflammatory response influences clinical outcome of ACS [14]. Peripheral leukocytes play an important role in the inflammatory response, interact with the endothelium in controlling vascular homeostasis, and are involved in the initiation of atherogenesis [15, 16]. In this context, this study is aimed at examining the proinflammatory effects of DEP on human peripheral blood mononuclear cells (PBMC) and the underlying mechanisms using THP-1 cells.

## 2. Materials and Methods

### 2.1. Reagents

5-(and-6)-Carboxy-2′,7′-dichlorodihydrofluorescein diacetate (carboxy-H2 DCFDA) was purchased from Invitrogen Corporation (USA). The antioxidant N-acetyl-L-cysteine (NAC) was obtained from Sigma Company (USA). The selective EGFR inhibitor, PD153035, was procured from Aladdin Industrial Corporation (Shanghai, China). RPMI1640 medium and RIPA lysis buffer were obtained from Beijing Solarbio Life Sciences Company (China). Lactate dehydrogenase (LDH) assay kit was obtained from Jiangsu Beyotime Biotechnology, Inc. (China). Interleukin 8 (IL-8) and tumor necrosis factor *α* (TNF*α*) ELISA assay kits were purchased from Wuhan Boster Bioengineering, Inc. (China). The rabbit antibodies against phospho-EGFR (Y1068) and pan-EGFR antibodies were obtained from Cell Signaling Technology (Beverly, MA, USA). Horseradish peroxidase (HRP)-conjugated goat anti-rabbit antibody was purchased from Santa Cruz Biotechnology (Santa Cruz, CA, USA).

### 2.2. Study Subjects

The study subjects were recruited from December 2014 to June 2015 in the 4th Affiliated Hospital of Xinxiang Medical University. All 25 patients (15 males and 10 females) were diagnosed of ACS with coronary angiography, indicating that more than one branch of coronary arteries blocked with an area of ≥70%. The subjects were aged 25 to 76 years with a mean age of 57.6 ± 11.5. This study was conducted in accordance with the Declaration of Helsinki, and the protocol was approved by the Ethics Committee of Xinxiang Medical University. Written informed consent was obtained from all subjects.

### 2.3. Cell Culture

Blood was drawn with venepuncture from a large antecubital vein. PBMC were purified using lymphocyte separation medium following the manufacturer's instruction. THP-1 cell line, a human leukemia mononuclear cell line, that has been extensively used to study monocyte/macrophage functions, mechanisms, signaling pathways, and nutrient and drug transport [17], was purchased from American Type Culture Collection (Manassas, VA, USA). Purified PBMC or THP-1 cells were cultured in RPMI 1640 medium containing 10% FBS with penicillin (100 U/ml) and streptomycin (100 *μ*g/ml) at 37°C in 5% CO_2_; 1 × 10^6^ cells in 0.5 ml medium were incubated in round-bottom polystyrene culture tubes with loosened caps for DEP stimulation studies.

### 2.4. DEP Preparation

DEPs used in this study were a generous gift from the Division of Environmental Public Health, United States Environmental Protection Agency. The manufacture and characteristics of the DEPs were depicted previously [18].

DEPs stored in the glass sample jar were suspended in molecular grade water to make a stock solution of 1 mg/ml and sonicated just before incubation with PBMC or THP-1 cells. The particle size of DEP was less than 0.45 *μ*m.

### 2.5. Enzyme Linked Immunosorbent Assay (ELISA)

1 × 10^6^ PBMC and THP-1 cells in 0.5 ml of RPMI 1640 medium were incubated with DEP at 37°C for 24 h, respectively. The culture tubes were centrifuged at 500 g for 10 minutes. Levels of IL-8 and TNF*α* proteins in the supernatants of culture medium were measured by ELISA, respectively, following the manufacturer's instruction. The phosphate-buffered saline (PBS) solution was used as a negative control.

In addition, THP-1 cells were pretreated with 10 mM NAC or 10 *μ*M PD153035, a selective EGFR inhibitor, for 2 h, respectively, prior to stimulation with 100 *μ*g/ml DEP for another 24 h. Levels of IL-8 and TNF*α* were measured with ELISA.

### 2.6. Measurement of Intracellular ROS

The intracellular formation of ROS in THP-1cells was detected using the fluorescent ROS probe carboxy-H2DCFDA. The intensity of green fluorescence produced by THP-1 cells is proportional to the amount of ROS produced. Briefly, THP-1 cells were preincubated with 20 *μ*M carboxy-H2DCFDA at 37°C for 1 h before exposure to 0, 10, 50, or 100 *μ*g/ml DEP for 4 h. The cells were washed once with PBS, suspended in 0.5 ml PBS, and put on ice before determining the green fluorescence intensity. Flow cytometry was performed with an Accuri C6 cytometer (Becton-Dickinson, USA). 6,000 events were counted for all sample runs. Relative cell size and density/granularity were quantified by analyzing light scatter properties, namely forward scatter for cell size and side scatter for density/granularity, and recording the mean fluorescence intensities (MFI) for each.

In addition, THP-1 cells were preincubated with 20 *μ*M carboxy-H2DCFDA for 1 h before further treatment with 10 mM NAC for 2 h. Then, 100 *μ*g/ml DEPs were added and incubated for another 4 h. Intracellular ROS levels were measured as described previously [19].

### 2.7. Immunoblotting

THP-1 cells exposed to 0, 10, 50, or 100 *μ*g/ml DEP for 4 h were washed twice with ice-cold PBS and then lysed in RIPA buffer (1x PBS, 1% nonidet P-40, 0.5% sodium deoxycholate, 0.1% sodium dodecyl sulfate (SDS), and 1 mM phenylmethylsulfonyl fluoride). The supernatants of cell lysates were subjected to SDS-PAGE. Proteins were transferred onto nitrocellulose membrane. Membrane was blocked with 5% nonfat milk, washed briefly, and incubated with primary antibody (1 : 1000 dilution) against phospho-specific or pan-EGFR at 4°C overnight, respectively, followed by incubation with corresponding HRP-conjugated secondary antibody (1 : 2000 dilution) for 1 h at room temperature. Immunoblot images were detected using ECL reagents and the Chemiluminescent Imaging System (Tanon Science & Technology Co., Shanghai, China).

### 2.8. Statistical Analysis

Data are presented as mean ± SD and analyzed with SPSS17.0 software. Data comparisons were carried out using one-way analysis of variance followed by Dunnett's posttest and two-tailed Student's *t*-test, with the overall *α* level set at 0.05.

## 3. Results

### 3.1. DEP Exposure Increases Expression of Proinflammatory Mediators

We first examined the proinflammatory effect of DEP on the PBMC from ACS patients. Exposure of the PBMC to 10-100 *μ*g/ml for 24 h did not result in significant alterations in cytotoxicity, as assessed by assay of LDH activity released into the culture medium (data not shown).

**Figure 1:**
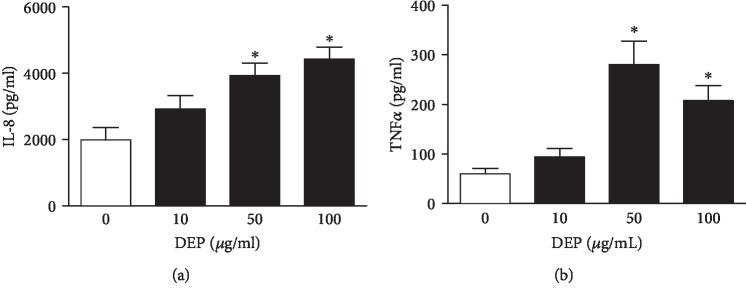
DEP exposure induces overexpression of proinflammatory mediators in human PBMC. The PBMC from ACS patients were incubated with 10-100 *μ*g/ml DEP for 24 h, respectively. The supernatants of cell culture media were collected through centrifuge. Levels of IL-8 (a) or TNF*α* (b) were measured using ELISA. ^∗^*P* < 0.05, compared with 0 *μ*g/ml of DEP, *n* = 25.

Under the same exposure condition, DEP treatment increased the expression of proinflammatory mediators including IL-8 and TNF*α* in the PBMC from ACS patients. As shown in Figure 1(a), DEP exposure increased IL-8 expression in a concentration-dependent fashion (*P* < 0.05). With the increase in the concentration of DEP, levels of TNF*α* in culture media reached the highest at 50 *μ*g/ml of DEP (*P* < 0.05) (Figure 1(b)). These results indicated that DEP stimulation increased the expression of proinflammatory mediators in human PBMC from ACS patients.

**Figure 2:**
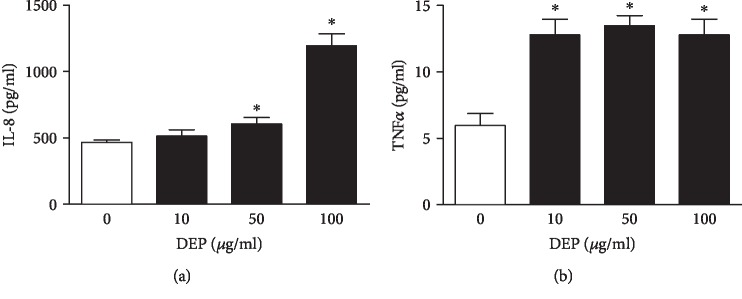
DEP exposure induces overexpression of proinflammatory mediators in THP-1 cells. THP-1 cells were incubated with 10-100 *μ*g/ml DEP for 24 h, respectively. The supernatants of cell culture media were collected through centrifuge. Levels of IL-8 (a) or TNF*α* (b) were measured using ELISA. ^∗^*P* < 0.05, compared with 0 *μ*g/ml DEP, *n* = 3.

DEP-induced expression of proinflammatory mediators was also examined in THP-1 cell line. First of all, we determined the proinflammatory effect of DEP on THP-1 cells under the same experimental conditions as the PBMC from ACS patients. As shown in Figure 2, exposure to DEP (10, 50, and 100 *μ*g/ml) induced a concentration-dependent increase in IL-8 (a) and TNF*α* (b) expression. Overall, THP-1 cells exposed to DEP presented similar proinflammatory response to the PBMC from ACS patients. To minimize the background interference of the PBMC from different ACS patients, we just used THP-1 cells, instead of the PBMC from ACS patients, to study the potential signaling pathways that may regulate DEP-induced cytokine release.

### 3.2. Oxidative Stress Mediates DEP-Induced Expression of Proinflammatory Mediators in THP-1 Cells

**Figure 3:**
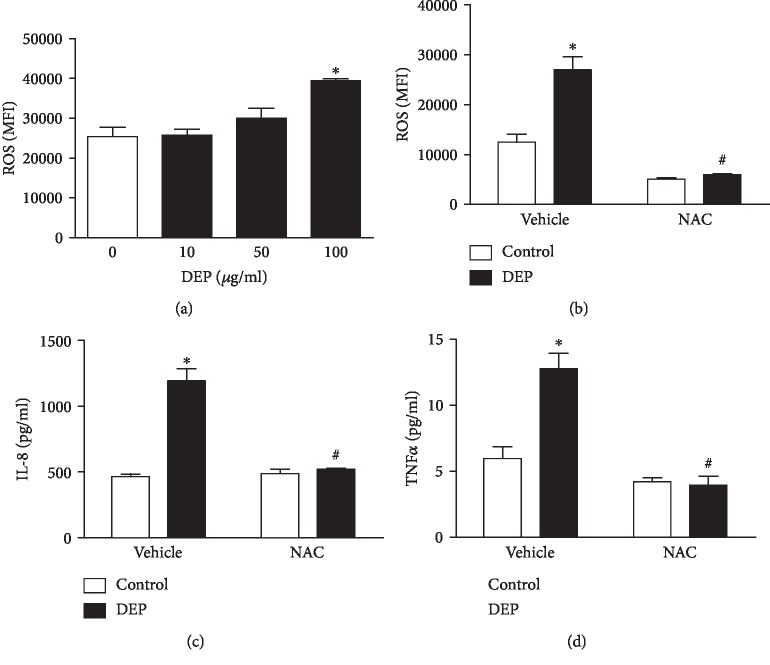
Oxidative stress is required for DEP-induced expression of IL-8 and TNF*α* in THP-1 cells. (a) THP-1 cells were treated with 10-100 *μ*g/ml DEP for 4 h, and intracellular ROS levels were measured using the fluorescent probe carboxy-H2 DCFDA and flow cytometer. (b) THP-1 cells were pretreated with 10 mM NAC for 2 h, prior to DEP (100 *μ*g/ml) treatment for another 4 h. ROS was detected as described previously. Furthermore, THP-1 cells were pretreated with 10 mM NAC for 2 h, prior to DEP treatment for another 24 h. Levels of IL-8 (c) and TNF*α* (d) were measured using ELISA, respectively. ^∗^*P* < 0.05, compared with vehicle control, *n* = 3. ^#^*P* < 0.05, compared with vehicle DEP, *n* = 3.

To determine whether oxidative stress is involved in DEP-induced inflammatory response in human blood mononuclear cells, we first measured intracellular levels of ROS in THP-1 cells, an indicator of oxidative stress. ROS levels were detected using flow cytometry and represented as MFI. As shown in Figure 3(a), exposure to DEP (10-100 *μ*g/ml) increased ROS levels in THP-1 cells, indicating that DEP stimulation caused oxidative stress. As expected, DEP-induced ROS production were blocked by the antioxidant NAC (Figure 3(b)). These results indicated that DEP induced oxidative stress in human blood mononuclear cells.

Furthermore, THP-1 cells were pretreated with the antioxidant NAC for 2 h prior to stimulation with 100 *μ*g/ml DEP for another 24 h. As shown in Figures 3(c) and 3(d), pretreatment of THP-1 cells with NAC significantly blocked DEP-induced IL-8 and TNF*α* expressions, respectively, indicating that oxidative stress was indispensable to DEP-induced release of proinflammatory mediators from human mononuclear cells.

### 3.3. EGFR Regulates DEP-Induced IL-8 Expression in THP-1 Cells

**Figure 4:**
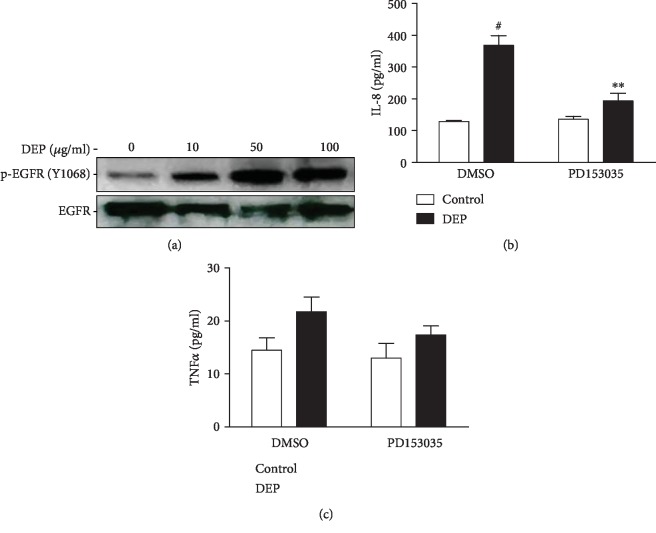
The EGFR is required for DEP-induced IL-8 expression in THP-1 cells. (a) THP-1 cells were treated with 10-100 *μ*g/ml DEP for 4 h. Cells were lysed, and the supernatants of cell lysates were subjected to SDS-PAGE and immunoblotting using phospho and pan antibodies against EGFR. (b, c) THP-1 cells were pretreated with 10 *μ*M PD153035 for 2 h before further treatment with 100 *μ*g/ml DEP for 24 h. Levels of IL-8 and TNF*α* were measured using ELISA. ^∗^*P* < 0.05, compared with vehicle control, *n* = 3. ^#^*P* < 0.05, compared with vehicle DEP, *n* = 3. Data shown are representative of three separate experiments.

To examine whether the EGFR is required for DEP-induced proinflammatory response in THP-1 cells, phosphorylation of EGFR at a major autophosphorylation tyrosine 1068 was first determined. The phosphorylation of EGFR is an indicator of its activation. As shown in Figure 4(a), exposure to DEP (10-100 *μ*g/ml) resulted in a concentration-dependent increase in EGFR phosphorylation at tyrosine 1068, indicating that the EGFR was activated by DEP treatment. The pretreatment of THP-1 cells with the selective EGFR inhibitor PD153035 markedly blocked DEP-induced expression IL-8 (Figure 4(b)), but not TNF*α* (Figure 4(c)).

### 3.4. Oxidative Stress Regulates EGFR Activation in THP-1 Cells Exposed to DEP

**Figure 5:**
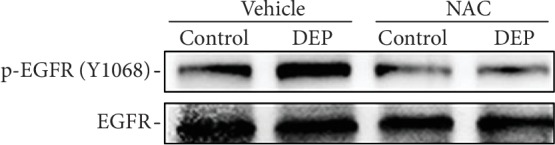
Oxidative stress regulates DEP-induced EGFR activation in THP-1 cells. THP-1 cells were pretreated with 10 mM NAC for 2 h, prior to 100 *μ*g/ml DEP treatment for 4 h. Then, the treated THP-1 cells were lysed, and the supernatants of cell lysates were subjected to SDS-PAGE and immunoblotting using phospho and pan antibodies against EGFR. Data shown are representative of three separate experiments.

As depicted previously, DEP treatment induced oxidative stress and EGFR activation. To further explore the link between these two events, THP-1 cells were pretreated with 10 mM NAC for 2 h, prior to 100 *μ*g/ml DEP treatment for 4 h. As shown in Figure 5, the pretreatment of THP-1 cells with NAC significantly blunted DEP-induced EGFR activation, indicating that oxidative stress was required for DEP-induced EGFR activation.

## 4. Discussion

Exposure to air pollution, especially PM, is associated with heart disease, even at the relatively low concentrations [5]. These associations are the strongest for PM_2.5_ [3], of which the combustion-derived nanoparticulates of diesel exhaust are an important component [2]. ACS include unstable angina and myocardial infarction. In both conditions, the coronary artery blood flow is impaired due to arteriosclerosis or thrombosis. Local and systemic inflammation has been proposed to play a pathogenic role in the pathogenesis of ACS [14]. In this present study, we employed the *in vitro* model to examine the proinflammatory effect of DEP on the PBMC from ACS and the underlying mechanisms using THP-1 cells. It was found that exposure to DEP induced overexpression of proinflammatory mediators in human mononuclear cells and this process was regulated by oxidative stress and EGFR.

Oxidative stress is a disturbance in the oxidant-antioxidant balance in favor of the former, leading to potential damage [20]. ROS, such as superoxide, hydrogen peroxide, and hydroxyl radicals, are a major contributor to oxidative stress and arise not only from the redox potential of the pollutants themselves but also from the circulating cytokines and inflammatory cells that are activated by inhaled PM or its soluble components [21]. Previous studies have showed that exposure to PM_2.5_ or DEP induces ROS production in human bronchial epithelial cells, resulting in overexpression of proinflammatory mediators [19, 22, 23]. In this study, we detected overt ROS production in DEP-treated blood mononuclear cells, and this event was required for DEP-induced TNF*α* and IL-8 expressions. The possible mechanisms for DEP-induced ROS production are under speculation. DEPs are typically composed of carbon particles and organic and inorganic compounds, such as polycyclic aromatic hydrocarbons (PAHs), nitro and oxygenated derivatives of PAHs, heterocyclic compounds, aldehydes, aliphatic hydrocarbons, and heavy metals. It has been demonstrated that the two main organic compounds adsorbed on DEP, PAHs, and quinones contribute to ROS production through enzymatic or nonenzymatic reactions [24, 25]. For example, PAHs can induce oxidative stress indirectly through biotransformation by cytochrome P450 and dihydrodiol dehydrogenase [26].

In addition to showing the involvement of oxidative stress in DEP-induced TNF*α* and IL-8 expression, this study also revealed that the EGFR regulated DEP-induced IL-8 expression in THP-1 cells. The EGFR is a single transmembrane protein that possesses intrinsic tyrosine kinase activity. It can be directly activated or transactivated through phosphorylation of autophosphorylation sites including tyrosine 1068 (Y1068) on its C-terminus in response to a variety of environmental stimuli [27]. The EGFR signaling cascade represents a key intermediate pathway involved in the regulation of proinflammatory genes and appears to be activated and upregulated in the lungs of human volunteers exposed to diesel exhaust [28]. Previous studies have demonstrated that PM- or PM component-mediated EGFR signaling plays a critical role in inducing the proinflammatory response in human airway epithelial cells [29, 30]. In this study, we observed that DEP stimulation caused a dose-dependent increase in EGFR phosphorylation in THP-1 cells, further upregulating DEP-induced IL-8 expression. As for the mechanisms of DEP-induced EGFR activation, it has been shown that the nonreceptor tyrosine kinase Src and the matrix metalloproteinase-mediated release of heparin-binding EGF regulated DEP- and metal-induced EGFR activation [31, 32]. Another study also with human bronchial epithelial cells has found that expression of IL-8 in response to DEP treatment involves autocrine production of EGFR ligands [33]. Additionally, airborne particles and chemicals adhered can interfere with membrane properties by changing membrane fluidity and composition of microdomains, further affecting activity of transmembrane proteins including receptors [34]. PAHs in DEP have been reported to increase the fluidity of cellular model membranes directly, and benzo[a]pyrene may interact with carbonyl groups of phospholipids [35]. Moreover, DEP-induced EGFR phosphorylation may involve protein tyrosine phosphatase (PTP) since in the previous study with primary human airway epithelial cells, we observed that DEP-induced EGFR activation is related to a loss of PTP activities which normally function to dephosphorylate EGFR in opposition to baseline EGFR kinase activity [36].

A previous study with human airway epithelial cells showed that pretreatment with an antioxidant prevented DEP-induced activation of EGFR [37], implying that oxidative stress induced by DEP may play a critical role in activating EGFR signaling. The association of oxidative stress with EGFR activation in DEP-treated mononuclear cells was examined in this study. It was revealed that oxidative stress was involved in the regulation of EGFR activation induced by DEP. Documented evidence indicates that ROS transiently inactivates PTPs to enhance or prolong EGFR activation [38]. Such redox-mediated PTP inactivation is due to the oxidation of a susceptible conserved catalytic cysteine residue that is essential for phosphotyrosine hydrolysis [39]. How EGFR is activated following DEP-induced oxidative stress in this study remains to be elucidated.

In this study, the mechanisms underlying DEP-induced proinflammatory effect were conducted using THP-1 cells. Although both THP-1 cells and primary human PBMC are categorized as human mononuclear cells, they still showed differential profile of proinflammatory mediators after DEP treatment. For example, exposure to DEP increased both IL-8 and TNF*α* releases from the PBMC. In contrast, only IL-8 but not TNF*α* release was detected in DEP-treated THP-1 cells. In other studies with human and murine alveolar macrophages, DEP treatment suppresses TNF*α* response [40–42]. Intriguingly, the DEPs generated from different sources showed minimal effect on IL-8 and C-C chemokine ligand 2 expression in THP-1cells [43]. Whether these apparent discrepancies in the proinflammatory potential of biodiesel are due to cell specific effects or differences in the chemical composition of the DEP used in the different studies remains to be clarified.

## 5. Conclusions

Diesel engine emissions are among the most prevalent anthropogenic pollutants worldwide. Exposure to combustion-derived PM air pollution has been related to increased incidence of cardiovascular morbidity and mortality, specifically in susceptible populations. Previous studies have demonstrated that ACS and other thrombotic effects are associated with acute exposure to DEP [4, 44]. Furthermore, the results from this *in vitro* study show that exposure to DEP induces inflammatory effects in human mononuclear cells and this process is regulated by oxidative stress and EGF receptor. Therefore, this study not only provides evidence for the elucidation of mechanisms underlying DEP-induced ACS exacerbation but also provide clues for the design of preventive and therapeutic measures against DEP-induced cardiovascular toxicity.

## Data Availability

The data used to support the findings of this study are available from the corresponding author upon request.
